# Hederagenin Supplementation Alleviates the Pro-Inflammatory and Apoptotic Response to Alcohol in Rats

**DOI:** 10.3390/nu9010041

**Published:** 2017-01-06

**Authors:** Gyeong-Ji Kim, Da Hye Song, Han Seok Yoo, Kang-Hyun Chung, Kwon Jai Lee, Jeung Hee An

**Affiliations:** 1 Division of Food Bioscience, Konkuk University, Chunju 27478, Korea; kgj8495@hanmail.net (G.-J.K.); sdh5740@naver.com (D.H.S.); 2 Department of Food Science and Technology, Seoul National University of Science & Technology, Seoul 01811, Korea; yhs0223@naver.com (H.S.Y.); carl@seoultech.ac.kr (K.-H.C.); 3 Department of Advanced Materials Engineering, Daejeon University, Daejeon 34520, Korea; jmul@ssu.ac.kr

**Keywords:** alcohol, hederagenin, liver disease, ALDH2, inflammatory, apoptosis

## Abstract

In this study, we determined the effects of hederagenin isolated from *Akebia quinata* fruit on alcohol-induced hepatotoxicity in rats. Specifically, we investigated the hepatoprotective, anti-inflammatory, and anti-apoptotic effects of hederagenin, as well as the role of AKT and mitogen-activated protein kinase (MAPK) signaling pathways in ethanol-induced liver injury. Experimental animals were randomly divided into three groups: normal (sham), 25% ethanol, and 25% ethanol + hederagenin (50 mg/kg/day). Each group was orally administered the respective treatments once per day for 21 days. Acetaldehyde dehydrogenase-2 mRNA expression was higher and alcohol dehydrogenase mRNA expression was lower in the ethanol + hederagenin group than those in the ethanol group. Pro-inflammatory cytokines, including TNF-α, IL-6, and cyclooxygenase-2, significantly increased in the ethanol group, but these increases were attenuated by hederagenin. Moreover, Western blot analysis showed increased expression of the apoptosis-associated protein, Bcl-2, and decreased expression of Bax and p53 after treatment with hederagenin. Hederagenin treatment attenuated ethanol-induced increases in activated p38 MAPK and increased the levels of phosphorylated AKT and ERK. Hederagenin alleviated ethanol-induced liver damage through anti-inflammatory and anti-apoptotic activities. These results suggest that hederagenin is a potential candidate for preventing alcoholic liver injury.

## 1. Introduction

Recently, adolescent alcohol consumption has been associated with negative health and social consequences in Korea [1]. In addition, alcohol-related liver disease showed a major cause of morbidity and mortality worldwide [2]. Alcoholic liver disease is characterized by lipid accumulation, inflammation and apoptosis, leading to cirrhosis, fibrosis and liver cancer [3]. Several studies have shown that oxidative stress and acetaldehyde play an pivotal role in the pathogenesis of alcoholic liver disease, including hepatocyte dysfunction, inflammation, apoptosis, and fibrosis [1,2,3].

In the liver, ethanol is oxidized to a toxic form, acetaldehyde, by alcohol dehydrogenase (ADH). Acetaldehyde is then oxidized to acetic acid, which is non-toxic, by acetaldehyde dehydrogenase (ALDH) [4]. As a toxic by-product of ethanol metabolism, acetaldehyde has greater chemical reactivity and toxicity than ethanol [5]. Acetaldehyde can combine with proteins and form aldehyde protein adducts that lead to protein dysfunction and result in the creation of antigens that contribute to inflammation [6]. The elimination of acetaldehyde is regarded as an important process in the prevention of alcoholic liver disease [7].

Generally, chronic alcohol consumption elevates the production of pro-inflammatory cytokines [8,9]. Pro-inflammatory mediators are involved in inflammatory responses, and of these, inducible interleukin-6 (IL-6), cyclooxygenase-2 (COX-2) and tumor necrosis factor (TNF-α) play important roles [10]. In particular, TNF-α can bind to its corresponding membrane receptor and further increase the generation of reactive oxygen species (ROS), contributing to the development of alcoholic liver disease and induction of apoptosis [4].

Apoptosis is a gene-regulated phenomenon that occurs through several pro- and anti-apoptotic genes expressing homologous proteins of the Bcl-2 family, such as Bcl-2 and Bax, which are known to play a major role in determining whether a cell undergoes apoptosis [11]. Previously, it was shown that chronic ethanol-induced apoptosis increases the expression of p53 and the molecular Bax/Bcl-2 ratio [12]. Especially, the tumor suppressor protein p53 is activated by DNA damage- or oncogene-induced signaling pathways and promotes the transcription of several genes that are involved in apoptosis, including those encoding death receptors and pro-apoptotic members of the Bcl-2 family [13]. In most cases, p53-induced apoptosis promotes release of cytochrome c in mitochondrial and induced caspase activation [14]. Under normal conditions, Bcl-2 levels are maintained in the cell; however, following a toxic stimulus, Bcl-2 initiates apoptosis [11]. A decrease in Bcl-2 protein expression results in the release of the apoptotic protein, Bax [15]. The Bax/Bcl-2 ratio has been used as an important marker for ethanol-induced apoptosis [11].

Hederagenin is a pentacyclic triterpenoid saponin that acts as a chemotaxonomic marker for plants of the Sapindaceae family [16]. In addition, multiple pharmacological activities have been attributed to hederagenin, including anti-hyperlipidemic, anti-lipid peroxidation, anti-platelet aggregation, hepatoprotective, and anti-inflammatory properties [16]. Hederagenin also showed a protective effect on vascular walls by improving lipid metabolism disorders and lipid deposition [17]. Recently, hederagenin showed anti-edema effects [18] and induced autophagy and promoted the degradation protein in neurodegenerative disease [19]. However, the effects of hederagenin on alcoholic liver injury and the mechanisms underlying these responses remain unclear.

In the present study, a Wistar rat model of alcoholic liver disease was established to evaluate the beneficial effects of treatment with hederagenin against alcoholic liver damage, including its anti-inflammatory and anti-apoptotic effects. We report for the first time the effects of hederagenin isolated from the *Akebia quinata* fruit on apoptotic and cytokine pathways associated with alcohol exposure.

## 2. Materials and Methods

### 2.1. Cell Culture

RAW 264.7 cells (Korea Cell Line Bank, Seoul, Korea) were cultured in Dulbecco’s modified Eagle’s medium (DMEM) containing 10% fetal bovine serum (FBS, Hyclone, Logan, UT, USA) and 1% penicillin–streptomycin (GIBCO, Grand Island, NY, USA) in a 5% CO_2_ incubator at 37 °C.

### 2.2. Akebia Quinata (AQ) Extraction and Isolation of Hederagenin

**Figure 1:**
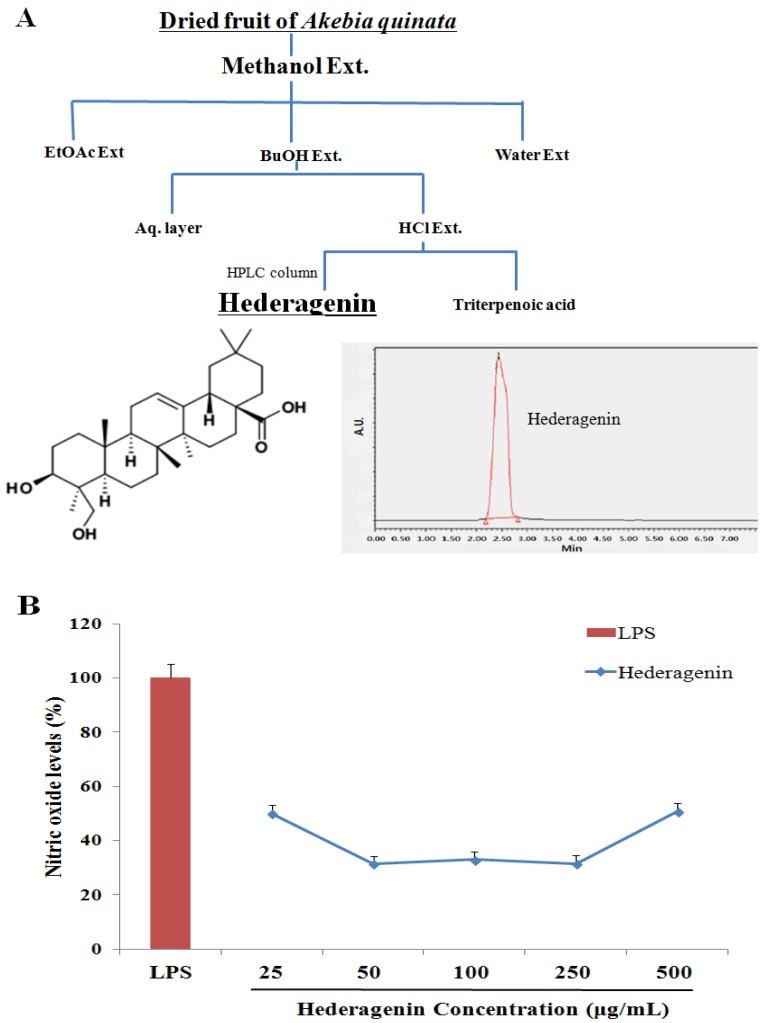
Flows diagram of extraction and isolation of hederagenin from *Akebia quinata* (**A**); and suppressive effect of hederagenin against Nitric oxide generation in RAW 264.7 cells (**B**).

Whole fruit of *Akebia quinata* cultivated in Jirisan (Hamyang-gun, Gyeongsangnam-do) was purchased from gyeongdong market (Seoul, Korea). The air-dried and milled fruits of *Akebia quinata* (4 kg) were subjected to extraction with methanol (40 L) for up to 24 h at 23 °C [20]. The filtered extract was concentrated under a vacuum to yield 400 g of residue, which was dissolved in methanol and sequentially partitioned using ethyl acetate, n-butanol, and water for 24 h at 25 °C in a shaking incubator. After filtration through filter paper (Whatman #2), the n-butanol-soluble fraction (20 g) was hydrolyzed in 5% HCl in MeOH: H_2_O (2:8 v/v) under reflux for 4 h. After cooling, the reaction mixture was extracted with ethyl acetate. The ethyl acetate-soluble fraction (0.3 g) was washed with distilled water and subjected to thin layer chromatography (TLC) and EtoAC: MeOH: H_2_O (70:27:3 v/v/v) to produce three sub-fractions (Figure 1A). High-performance liquid chromatography (HPLC) with a reverse-phase column (SunFire C18, 4.6 × 250 mm, 5-μm diameter; Waters, Milford, MA, USA) and HPLC Empower Software (Waters, Milford, MA, USA) were used to analyze the compounds in the extract. The mobile phase was acetonitrile: methanol: water. The flow rate was 1 mL/min, and the injection volume was 20 μL. The chromatograms were detected at 270 nm and collected at 30 °C. Hederagenin was purchased from Sigma-Aldrich (St. Louis, MO, USA) and used as an authentic standard (Figure 1A).

### 2.3. Nitric Oxide (NO) Assay

RAW 264.7 cells (1 × 10^6^ cells/well) were cultured in 96-well plates and incubated at 37 °C for 24 h [21]. The medium was then removed from each well and replaced with phenol red-free DMEM. For assays incorporating the various treatments, cells were first activated by the addition of lipopolysaccharide (LPS, 1 mg/mL), tetrahydrobiopterin (BH_4_, 10 μg/mL), 200 mM l-arginine, and interferon-γ (IFN-γ, 100 U/mL) for 24 h at 37 °C and 5% CO_2_. Cells with media alone served as a negative control and activated cells served as a positive control. In the presence of NO, the Griess reagent forms a violet color. Therefore, the supernatant from each well was transferred to a fresh 96-well plate and mixed with Griess reagent (1% sulfanilamide and 0.1% naphthylethylene diamine dihydrochloride in 2% H_3_PO_4_) for 10 min at room temperature. The optical density of the samples was obtained using a spectrophotometer (Biochrom) at 540 nm. Cell viability was assessed using the MTT assay.

### 2.4. Animal Experiments

All of the experiments were performed with approval from the Institutional Animal Care and Use Committee at Konkuk University (IACUC approval number KU 15057), Seoul, Republic of Korea. Male Wistar rats weighing 200 g and aged 5–6 weeks (Orient bio. Korea) were used in this study. The animals were maintained in temperature-controlled (21–22 °C) and light-controlled (12-h light, 12-h dark cycle) environments with 70% humidity and given free access to water and food. The experimental animals were randomly divided into three groups: (1) normal (sham); (2) 25% ethanol; and (3) 25% ethanol + hederagenin. During the 21-day experimental period, the rats were orally administered 1 mL of 25% ethanol or 1 mL of water (sham group). After administration of the ethanol or water, hederagenin was orally administered (50 mg/kg) according to the respective treatment groups. Treatment consumption was measured daily and weight gain was measured weekly. At the end of the 21-day period, the rats were fasted for 16 h and then killed by decapitation. Blood samples were collected from the heart, and the serum was separated by centrifugation at 1610× *g* for 30 min. The liver, kidney, and spleen were excised, and the blood and debris were removed by washing with physiological saline. The dry weight of the samples was recorded, and then they were stored at −80 °C until further analysis.

### 2.5. Biochemical Assays

Serum alanine aminotransferase (ALT) and aspartate aminotransferase (AST) activities were quantified to assess hepatotoxicity according to the methods described by Reitman and Frankel [22]. Total serum cholesterol levels were determined using commercial kits (Sigma, St. Louis, MO, USA) based on modification of the cholesterol oxidation method of Alain et al. [23]. Serum triglyceride concentrations were measured enzymatically using the Free Glycerol Determination Kit according to manufacturer’s instructions (Sigma, St. Louis, MO, USA).

### 2.6. Enzyme-Linked Immunosorbent Assay (ELISA) of TNFα and IL6

TNF-α and IL-6 levels in the liver were determined using mouse TNF-α and IL-6 ELISA kits (Abcam, Cambridge, UK) according to the manufacturer’s protocol. The amount was expressed as pg/mg protein.

### 2.7. Reverse Transcription-PCR

The liver tissue was separated from total RNA using Trizol solution (Trizol, Invitrogen, Carlsbad, CA, USA). cDNA was synthesized using the first strand cDNA synthesis kit (18080-051, Invitrogen, Carlsbad, CA, USA). PCR was performed using the KAPA Taq Extra PCR kit (KR0355, Kapa Biosystems, Wilmington, DE, USA). Primer sequences were as follows: GAPDH: 5′-ATCCCATCACCATCTTCCAG-3′, 5′-CCTGCTTCACCACCTTCTTG-3′, ALDH2: 5′-GCTGTCAGCAAGAAAACATTCCCC-3′, 5′-CTTGTCAGCCCAGCCAGCATAATA-3′, ADH: 5′-ACCATCGAGGACATAGAA-3′, 5′-GTGGAGCCTGGGGTCAC-3′, TNF-α: 5′-GTAGCCCACGTCGTAGCAAA-3′, 5′-CCCTTCTCCAGCTGGAAGAC-3′, COX-2: 5′-CTGCATGTGGCTGATGTCATC-3′, 5′-AGGACCCGTCATCTCCAGGGTAATC-3′, IL-6: 5′-CAAGAGACTTCCAGCCAGTTC-3′, 5′-GAAACGGAACTCCAGAAGACC-3′. PCR was initiated at 95 °C for 3 min followed by 30 cycles at 95 °C for 30 s and 50–60 °C for 30 s. The number of cycles and annealing temperature for each primer pair were optimized. A final extension of 72 °C for 10 min was included. The amplified PCR products were subjected to electrophoresis at 100 V through 1.2% agarose gels for 40 min. A 100 bp DNA ladder was used as a molecular marker. The bands were visualized with ethidium bromide and analyzed using BandScan.

### 2.8. Western Blotting Analysis

The rat livers were lysed in ice-cold lysis buffer (RIPA, 20 mM Tris-HCl (pH 7.5), 150 mM NaCl, 1 mM ethylenediaminetetraacetic acid disodium salt (Na_2_EDTA), 1 mM ethylene glycol-bis(β-aminoethyl ether)-*N*,*N*,*N*′,*N*′-tetraacetic acid (EGTA), 1% NP-40, 1% sodium deoxycholate, 2.5 mM sodium pyrophosphate, 1 mM β-glycerophosphate, 1 mM sodium orthovanadate (Na_3_VO_4_), 1 μg/mL leupeptin, and 1 mM phenylmethylsulfonyl fluoride (PMSF, as a protease inhibitor). The membranes were then incubated with β-actin (Cell Signaling Technology, Beverly, MA, USA), Bcl-2 (Abcam, Cambridge, UK), Bax (Cell Signaling Technology), Bcl-X (Abcam, Cambridge, UK), p53 (Abcam, Cambridge, UK), *p*-AKT (Cell Signaling Technology), *p*-ERK (Cell Signaling Technology), and *p*-p38 (Cell Signaling Technology) antibodies, followed by a goat anti-rabbit IgG (H+L) HRP-conjugated secondary antibody (Zymax, San Francisco, CA, USA). The blots were detected using chemiluminescence using an X-ray film (AGFA, Mortsel, Belgium).

### 2.9. Histopathological Observation

The rat liver and kidney were fixed with a 10% formaldehyde solution for 24 h, embedded in paraffin, and cut into 4-μm-thick slices. The slices were stained with hematoxylin–eosin (H&E) for routine histopathological examination, and then examined and imaged using a light microscope at ×100 magnification to determine the degree of hepatic steatosis.

### 2.10. Statistical Analysis

Statistical analysis was performed using SPSS 18.0 (SPSS Inc., Chicago, IL, USA). Averages and standard deviations were calculated and differences between groups were assessed using the analysis of variance method and the Duncan’s multiple range test. A difference was considered significant if *p* < 0.05.

## 3. Results

### 3.1. Inhibition of NO of Hederagenin from AQ

The data in the present study show a suppressive effect on NO generation following treatment with hederagenin attributable to inhibition of the de novo synthesis and catalytic activity of inducible nitric oxide synthase (iNOS) in RAW 264.7 cells. As shown in Figure 1B, no cytotoxicity was observed following treatment of the cells with hederagenin at concentrations of 50–500 μg/mL. Thus, the IC_50_ value by which hederagenin inhibited the formation of NO was determined to be 25 μg/mL (Figure 1B). In the present study, we show for the first time that hederagenin is an important phytochemical with the potential to scavenge free radicals and suppress the generation of NO in inflammatory leukocytes, including neutrophils and macrophages.

### 3.2. Weight Gain and Liver Weight

As shown in Table 1, weight gained in the 25% ethanol only and ethanol + hederagenin treated groups was slightly higher than that of the normal (sham) group, but the differences were not significant. Similarly, liver and kidney weights from the ethanol only and ethanol + hederagenin treated groups were lower than those of the normal group, but the differences were not significant (Table 1).

### 3.3. Effects of Hederagenin on Biomarkers of Liver Injury

AST and ALT levels in the ethanol-treated group were higher than those in the normal group; however, AST and ALT levels were lower in the ethanol + hederagenin group than those in the ethanol-treated group (Table 2). Total serum cholesterol levels were significantly higher in the ethanol-treated rats than those in the normal rats (Table 2), an effect that was suppressed by treatment with hederagenin. Triglyceride concentrations were also significantly higher in the ethanol-treated groups than those in the normal and ethanol + hederagenin-treated groups. However, total serum cholesterol and triglyceride levels were lower in the ethanol + hederagenin group than those in rats treated with ethanol. Therefore, hederagenin treatment reduced ALT, AST, total cholesterol, and triglycerides in the serum of rats.

### 3.4. Histological Analysis

**Figure 2:**
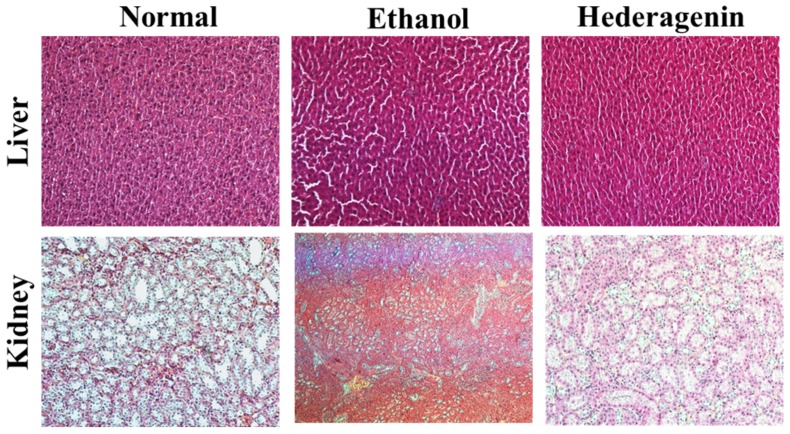
Histological features of representative liver and kidney sections stained with hematoxylin–eosin (H&E) after chronic ethanol consumption. Typical images were chosen from each experimental group (original magnification ×100).

Histological examination by H&E staining showed a normal liver lobular architecture in the control rats. However, the livers from rats administered ethanol showed micro- and macro-vesicular steatosis and excessive inflammatory cell infiltration. Those pathological changes were attenuated by hederagenin treatment (Figure 2). The liver of ethanol + hederagenin treated rats showed a similar pattern to the normal group. Our results suggest that hederagenin treatment attenuates the degree ethanol-induced liver fibrogenesis and inflammatory cell infiltration.

### 3.5. Effects of Hederagenin on Hepatic ADH and ALDH2 mRNA Expression

**Figure 3:**
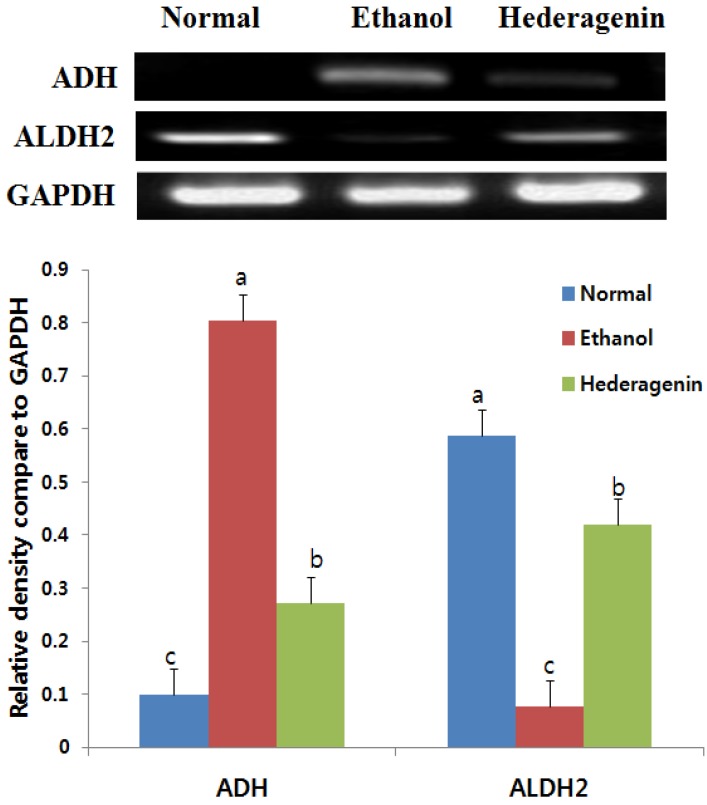
Effects of hederagenin on mRNA expression of ADH and ALDH2 assessed using RT-PCR. Results are expressed as the means ± SD. Significant differences (*p* < 0.05) are represented using different letters. ADH, alcohol dehydrogenase; ALDH, acetaldehyde dehydrogenase.

We investigated changes in the mRNA expression of ADH and ALDH2 using RT-PCR. Hepatic ADH mRNA expression levels were 8.09-fold higher in the ethanol-treated group than those in the normal group (Figure 3). However, ADH mRNA expression was 2.96-fold lower in the ethanol + hederagenin group than that in the ethanol-treated group. The level of ALDH2 mRNA expression was 7.68-fold lower in the ethanol group than that in the control group; however, this effect was partially attenuated in the ethanol + hederagenin group, which showed a 5.48-fold increase in ALDH2 mRNA expression over that in the ethanol-treated group. Thus, treatment with hederagenin increased the ethanol-induced suppression of ALDH2 mRNA expression.

### 3.6. Effect of Hederagenin on Inflammation

**Figure 4:**
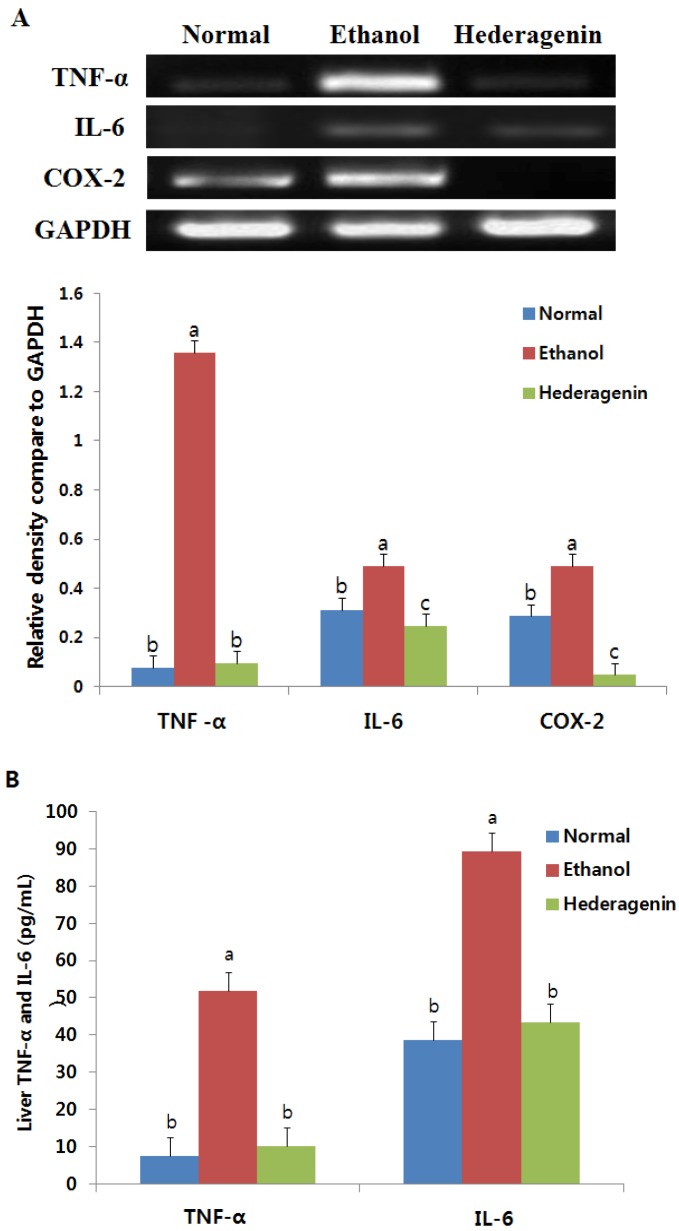
Effect of hederagenin on inflammation-related gene expression in the livers of ethanol-exposed rats (**A**); and mRNA expression of the liver TNF-α, IL-6, and COX-2 assessed using RT-PCR (**B**). The liver concentration of TNF-α and IL-6 was determined using an enzyme-linked immunosorbent assay. Results are expressed as the means ± SD. Significant differences (*p* < 0.05) are represented using different letters. TNF, tumor necrosis factor; IL, interleukin; COX, cyclooxygenase.

The level of TNF-α mRNA expression in the ethanol group was significantly higher (17.65-fold) than that of the normal group; however, TNF-α mRNA expression in the ethanol + hederagenin group was 14.49-fold lower than that of the ethanol-treated group (Figure 4A). COX-2 mRNA expression in the ethanol-treated group was markedly increased to 1.72-fold that of the normal group, and that increase was attenuated in the ethanol + hederagenin group. Similar to IL-6 mRNA expression levels significantly increased in the ethanol-treated group, but were reduced in the ethanol + hederagenin group. Hederagenin attenuated ethanol-induced increases in TNF-α, COX-2, and IL-6 mRNA expression.

The levels of TNF-α and IL-6 levels in the livers from control animals were 7.53 pg/mL and 38.70 pg/mL, respectively (Figure 4B). Ethanol consumption significantly increased the liver levels of TNF-α and IL-6 to 51.98 and 89.21 pg/mL, respectively. TNF-α levels in the ethanol + hederagenin group clearly decreased to 5.1-fold less than that in the ethanol-treated group. In addition, IL-6 levels in the ethanol + hederagenin group were reduced to 2.06-fold less than that in the ethanol-treated group. These results show the anti-inflammatory properties of hederagenin.

### 3.7. Effects of Hederagenin on the Expression of Bcl-2, Bax, Bcl-x, and p53

**Figure 5:**
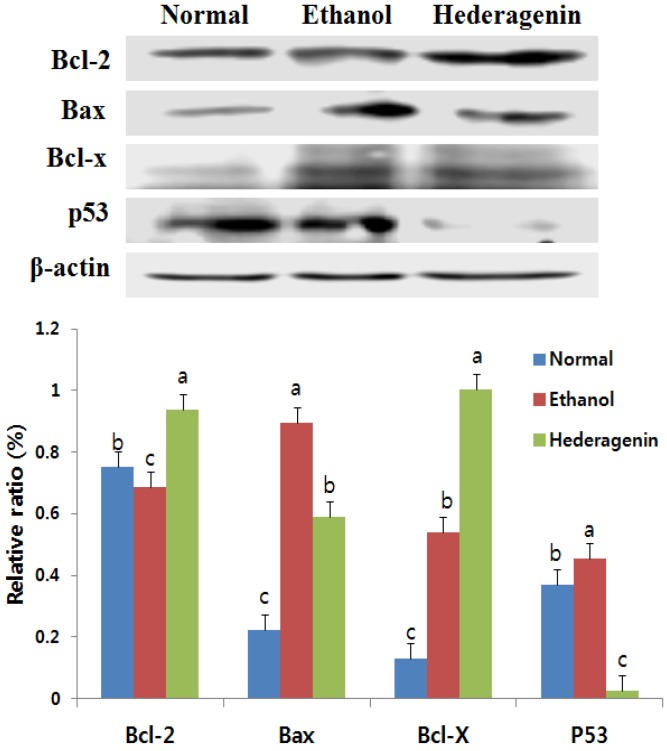
Effects of hederagenin on apoptotic signaling cascades in ethanol-treated hepatocytes. Extracts of the liver proteins from the different groups were subjected to Western blotting. Expression levels of cytoplasmic Bcl-2, Bax, Bcl-X, and p53 are shown. Results are expressed as the means ± SD. Significant differences (*p* < 0.05) are represented using different letters.

We examined the impact of hederagenin on apoptosis related protein expression in ethanol-induced liver injury by using Western blot analysis. Western blot analysis showed that p53 expression was activated in the ethanol group and clearly reduced in the ethanol + hederagenin group. As shown in Figure 5, expression of the anti-apoptotic protein Bcl-2 decreased, whereas expression of the pro-apoptotic protein Bax increased in the ethanol-induced hepatic injury. Further, the Bax/Bcl-2 ratio was elevated in the ethanol group. In contrast, treatment with hederagenin reversed the expression levels of Bcl-2 and Bax and reduced the Bax/Bcl-2 ratio. We further analyzed the expression of proapoptotic proteins in the liver tissue and determined that the expression of Bcl-xL was lower in the ethanol + hederagenin group than that of the ethanol group.

### 3.8. Effects of Hederagenin on Phosphorylation of AKT, ERK, and p38 MAPK

**Figure 6:**
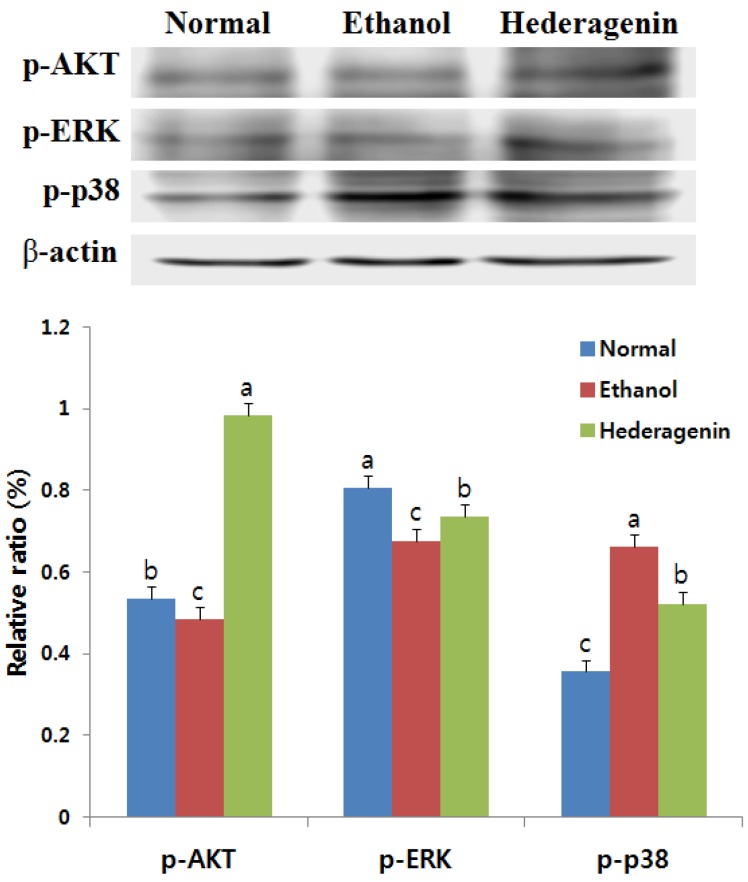
Effects of hederagenin on the expression levels of p-AKT, p-ERK, and p-p38 in the liver of ethanol-exposed rats. Results are expressed as the means ± SD. Significant differences (*p* < 0.05) are represented using different letters.

Hederagenin acts against ethanol-induced cytotoxicity by promoting AKT phosphorylation to form phospho-AKT (p-AKT) (Figure 6). We examined the effects of hederagenin on the activation of p38 MAPK and ERK pathways in ethanol-induced liver injury. Ethanol treated group increased the level of activated p38 MAPK, an effect that was blocked by the addition of hederagenin. Similarly, downregulation of activated p-ERK was observed in ethanol-treated rats, an effect that was increased by the addition of hederagenin.

## 4. Discussion

In the present study, supplementation of hederagenin for three weeks significantly inhibited the progression of alcoholic liver injury in rats. Hederagenin supplementation alleviated ethanol-induced liver injury and inflammation, as reflected by decreased serum AST, ALT, triglycerides, and total cholesterol levels, as well as decreased serum TNF-α and IL-6 levels. In addition, hederagenin reduced the inflammatory response to ethanol, as evidenced by significantly suppressed levels of hepatic TNF-α, IL-6, and COX-2 mRNA expression. Second, hederagenin supplementation modified the effects of ethanol on ADH and ALDH2, exerting a protective mechanism against ethanol-induced liver injury in rats. Third, hederagenin reduced apoptosis in the liver of rats exposed to ethanol, as shown by the decreased ratio of Bax/Bcl-2 and p53 activities. Finally, our study found that ethanol exposure reduces the expression of p-AKT and p-ERK. To the best of our knowledge, this is the first evidence that hederagenin reduces liver injury through anti-inflammatory and anti-apoptotic activities in rats exposed to ethanol.

Generally, ALT and AST levels are the most frequently used biomarkers for evaluating liver injury [4]. In this study, ALT and AST levels increased in the serum of ethanol-treated rats. However, this effect was attenuated by treatment with hederagenin. In addition, pro-inflammatory cytokines have emerged as important mediators of hepatic inflammation associated with excessive ethanol intake [3]. The release of inflammatory cytokines, such as TNF-α and IL-6, could lead to hepatocyte apoptosis [4]. Decreases in TNF-α, IL-6, and COX-2 activity have been used as valuable indicators of an inflammatory response to potentially toxic agents [3,6]. In our study, TNF-α and IL-6 levels in the livers from ethanol-treated rats were significantly increased. However, hederagenin treatment significantly attenuated the increased release of liver TNF-α and IL-6 levels observed in animals treated with ethanol. In addition, our study showed that expression of TNF-α, IL-6, and hepatic COX-2 mRNA was elevated after alcohol treatment. These increases were dramatically attenuated by hederagenin treatment. These results are in agreement with those of a study indicating that induction of TNF-α, IL-6, and COX-2 expression in rats administered ethanol was attenuated by *Agrimonia eupatoria* [3]. Those observations support data suggesting that pro-inflammatory cytokines are released by damaged hepatocytes, as well as the protective effect of hederagenin.

The ethanol metabolic pathway plays a significant role in the pathogenesis of alcoholic liver disease. Toxic acetaldehydes can lead to mitochondrial dysfunction and apoptosis, resulting in serious damage to liver function. Overexpression of ADH increases susceptibility to ethanol toxicity in myocardial cells. However, overexpression of ALDH has a protective effect on alcohol-induced cardiac injury [4,5,6,7]. In this study, we determined the mRNA expression of ADH and ALDH2. We found that hederagenin decrease ethanol-induced ADH activation. In addition, we observed that hederagenin activates ALDH2 in rats exposed to ethanol. These results suggest that the protective effects exerted by hederagenin may be attributable to modification of the ethanol metabolic pathway by preventing acetaldehyde accumulation.

Apoptosis is involved in the process of liver fibrosis. In addition, apoptosis may be used to modulate liver fibrosis [24]. Endogenous p53 activation in hepatocytes induced liver fibrosis [24]. Bcl-2 plays an important role in cell apoptosis [25]. The Bcl-2 family modulates apoptosis, with the Bax/Bcl-2 ratio serving as a rheostat to determine cell susceptibility to apoptosis [26]. In the present study, expression of Bcl-2 sharply decreased and the expression of Bax and p53 increased in ethanol-treated rats. In contrast, this tendency was reversed in the ethanol + hederagenin-treated animals. Our results show that hederagenin may be used as an anti-apoptotic agent.

The AKT, a serine/threonine kinase, is a key player in regulating cell signals that are important for cell death and survival. Activation of the AKT pathway promotes cell survival and is involved in the upregulation of Bcl-2 [27]. In this study, hederagenin treatment suppressed ethanol-induced reductions in activated AKT in the liver. In cultured hippocampal neurons and endothelial cells, elevations in activated ERK have been shown to mediate the inhibition of apoptosis, preventing the loss of activated ERK may be a mechanism by which hederagenin inhibits ethanol-induced apoptosis [26]. Our study shows that hederagenin treatment significantly attenuates the increase in activated p38 MAPK induced by ethanol. Hederagenin may inhibit ethanol-induced apoptosis by promoting the activation of AKT and ERK and blocking the activation of p38 MAPK.

In conclusion, the present study shows for the first time that hederagenin has various protective effects against liver injury in rats treated with ethanol. The protective activities of hederagenin in the liver against ethanol toxicity involve the reduction of acetaldehyde through the activation of ALDH2. Further, our study shows that hederagenin can protect against ethanol-induced liver injury by suppressing inflammatory mediators such as TNF-α, IL-6, and COX-2. Moreover, hederagenin decreases mediators of apoptosis (Bax and p53) by activating the AKT and ERK signaling pathways. Our findings suggest that hederagenin is a potential candidate for the prevention and treatment of ethanol-induced liver injury.

## Figures and Tables

**Table 1:** Effects of hederagenin from *Akebia quinata* fruit on body weight and liver weight in ethanol-treated rats.

Treatment	Normal	Ethanol	Hederagenin
Initial body weight (g)	128.5 ± 3.39 ^a^	128.71 ± 5.31 ^a^	130.17 ± 3.19 ^a^
Final body weight (g)	245.13 ± 41.49 ^a^	270.01 ± 31.01 ^a^	260.86 ± 9.68 ^a^
Body weight gain/day (g)	5.55 ± 1.81 ^a^	6.72 ± 1.22 ^a^	6.22 ± 0.31 ^a^
Liver weight (g)	10.90 ± 1.43 ^a^	9.61 ± 0.92 ^a^	9.39 ± 1.07 ^a^
Kidney weight (g)	2.61 ± 0.11 ^a^	2.44 ± 0.17 ^a^	2.34 ± 0.24 ^a^
LW/BW	0.045 ± 0.034 ^a^	0.036 ± 0.030 ^a^	0.036 ± 0.110 ^a^

Results are presented as mean ± standard deviation. Within rows, means with different superscripts are significantly different (*p* < 0.001).

**Table 2:** Effects of hederagenin from *Akebia quinata* fruit on serum aspartate aminotransferase (AST), alanine aminotransferase (ALT), total cholesterol (TC), and triglyceride (TG) levels in ethanol-treated rats.

Treatment	AST (IU/L)	ALT (IU/L)	TC (mg/dL)	TG (mg/dL)
Normal (*n* = 6)	113.03 ± 28.20 ^c^	35.14 ± 2.41 ^a^	85.55 ± 4.06 ^b^	30.28 ± 10.55 ^c^
Ethanol (*n* = 7)	235.93 ± 45.38 ^a^	42.17 ± 20.48 ^a^	95.47 ± 8.65 ^a^	55.32 ± 9.80 ^a^
Hederagenin (*n* = 6)	208.65 ± 32.94 ^b^	27.53 ± 7.38 ^a^	79.75 ± 5.24 ^b^	41 ± 9.79 ^b^

Results are presented as mean ± standard deviation. Within column, means with different superscripts are significantly different (*p* < 0.001).
